# Handling of life-threatening situations in the context of hazard control and emergency response: Master Medic/Master Physician – an academic course

**DOI:** 10.3389/fpsyt.2025.1718648

**Published:** 2025-11-25

**Authors:** Sabrina Ziehr, Philipp Hans Merkt

**Affiliations:** 1 Department of Disaster Prevention and Crisis Management (bzgk.de), Research and Education Center for Extraordinary Tactical Situations and Strategic Resilience (18_RECESS), University of Applied Sciences gem. Trägergesellschaft, Idstein, Germany; 2 Department of Human Medicine, Faculty of Health, University Witten Herdecke gGMBH, Witten, Germany

**Keywords:** resilience, Master Medic/Master Physician, curriculum, tactical emergency medicine, leadership

## Abstract

Hybrid threat or hazardous situations, particularly when life-threatening, are increasing nationally and internationally. They pose challenges for threat and hazard control and emergency response that require actors to have a comprehensive capability to act. In addition to a medical focus, also tactical aspects play a significant role in such situations. Combining these requirements is the objective of the Master Medic program, often understood as the most experienced medic in an unit, is expected to meet. A uniform concept in the sense of a curriculum at academic level is currently still lacking. The Department of Disaster Prevention & Crisis Management at the University of Applied Sciences Fresenius in Idstein, Germany, has designed the degree program in Tactical Emergency Medicine for Disaster Management and Counterterrorism (Master Medic/Master Physician) based on the diverse expertise of the police, military, and emergency service threat prevention sectors, as well as results from a survey of the potential target groups. The program also incorporates the concepts of Human Performance Optimization and Strategic Resilience (HPSR). As part of this program, graduates of the Master Medic or Master Physician program will be equipped to combine leadership, (tactical) operational medicine, training and continuing education, as well as research and development, in order to contribute significantly to the protection of the population, individuals, and cultural assets. This also corresponds to the NATO’s efforts, which defines strengthening resilience for defense and the Allied capability as a key development area.

## 1 Introduction

Hybrid threat situations (1) and critical environmental events are present worldwide. In this context, emergency services are increasingly confronted with life-threatening situations, which entail diverse consequences. In addition to an acute threat to people, material, and cultural assets across society (2), such situations require a high level of specialized coordination before, during, and after a particularly life-threatening event. The Sendai Framework for Disaster Risk Reduction (3), the Resilience Through Civil Preparedness Course (4) or the Resilience Reference Curriculum (5) highlight the need of structured programs to increase safety, stability and resilience in the society. Relevant interdisciplinary scientific approaches and models are incorporated into robust training in the context of strategic resilience (6). The tactical situation generally determines medical care (7). The primary goal always is to provide care for emergency patients to preserve their lives or prevent health damage. This is embedded in coordinated cooperation between the emergency personnel on site, usually consisting of the emergency services, fire department, and police, who jointly address the situation in a goal-oriented manner. The specific task should be accomplished with the available resources, which corresponds to the concept of human performance (8). Appropriate resilience among emergency personnel is also important for long-term resilience, as shown by various studies on the prevention of mental illness (9, 10). Emergency personnel with the most versatile and interdisciplinary training possible can represent a key control element for successfully managing of e. g. an hazardous situation. In the military context, this is the so-called Master Medic, who possesses comprehensive skills in medicine, strategy, management, and leadership.

## 2 Human performance and strategic resilience

The accomplishment of special operational situations requires appropriate human performance. In this context, the performance of the individual, as well as the performance of the entire team, is essential. In the military and sports, measures to increase performance are referred to as human performance augmentation, subdivided into human performance optimization and human performance enhancement (11). Examples for specific trainings in the field of human performance are the concept of total force fitness (12) or the Tactical Human Optimization, Rapid Rehabilitation and Reconditioning (THOR3) program (13). Both take place in the military setting and focus on preventing and training health-related behavior. A theoretical model that addresses human performance and its improvement is the model of strategic resilience (6). It argues that a well-coordinated interaction of physical, cognitive, and psychological factors can lead to improved operational outcomes by increasing resilience in special operational situations. In relation to threat prevention, this means that operational personnel must be in good physical condition (physical), has comprehensive knowledge and is skilled for appropriate decision-making (cognition). Furthermore, they have the necessary confidence in themselves, the team, and the ability to successfully handle the situation (psychological). This requires that they are well educated and trained to be able to draw on these skills in special operational situations. Education in the field of disaster medicine is mostly carried out using face-to-face lectures and simulation, sometime also via online lectures or e-learning (14). Most of the courses convey traditional disaster triage education and training and some integrate technologies like Augmented or Virtual Reality instead of simulation (15). Inter- or multisectoral collaboration is identified as a key point for improving the health workforce and handling the dynamic demands in public health (16).

## 3 The Master Medic as a professional profile

The title “Master Medic” is currently not clearly defined; however it designates the most experienced and professionally qualified medical specialist within a (military) unit. Nevertheless, there are clear training contents in the field of medicine, such as adhering to the principles of Tactical Combat Casualty Care (TCCC) to increase the survival rate (17). However, there is neither a general basic understanding of the Master Medic profession nor are there uniform educational training standards.

One occupational profile is, for example, the 18D of the U.S. Army Special Forces Medics. In 1994, Graham (18) was able to show in his analysis that a significant proportion of these highly specialized combat medics leave the unit after a few years to continue their medical careers in other occupational fields. A particularly common career aspiration is to become a physician assistant (PA) by studying – almost half of all respondents expressed an interest in this. A further 16–21% of SF medics aspire to study to become a medical doctor or train in teaching nursing professions (18). In addition to these specific follow-up positions, respondents cited other reasons for retirement: lack of institutional recognition of the SF 18D profile in military and civilian healthcare, insufficient opportunities for continuous professional development, and the perception of the job profile as a career cul-de-sac. Added to this is a negative perception of remuneration compared to other armed forces or academic professions, such as Navy Corpsmen or an PA (18). For the U.S. Army, this departure has a double significance: on the one hand, it loses operational specialists with practical expertise; on the other because of their further qualifications, it loses highly qualified officers who are no longer available to the military system.

Important conclusions can be made from these experiences for Europe and Germany: if a ‘Master Medic’ or ‘Master Physician’ is to be established as an academic career profile, clear career paths and follow-up opportunities must also be opened outside of a tactical job profile. These could include aspects such as leadership and management, but also qualifications for training and further education for civilian scenarios or research.

This has the potential to result in the loss of valuable know-how and skills. Particularly against the background of the desired academisation in various areas of the healthcare sector outside of conventional medicine, such as Therapy and Nursing (19) or the establishment of the professional field of Physician Assistant (20), an Academic, part-time education format presents itself as a beneficial way to anchor the competencies of the Master Medic in the curriculum. Training tactical, emergency and disaster medicine at an academic degree in Master of Science not only creates operational excellence, but also long-term career prospects – and thus sustainable retention of expertise in the interests of overall societal resilience (21).

## 4 The study program Tactical Emergency Medicine for Disaster Management and Counterterrorism – Master Medic/Master Physician in Germany

**Figure 1:**
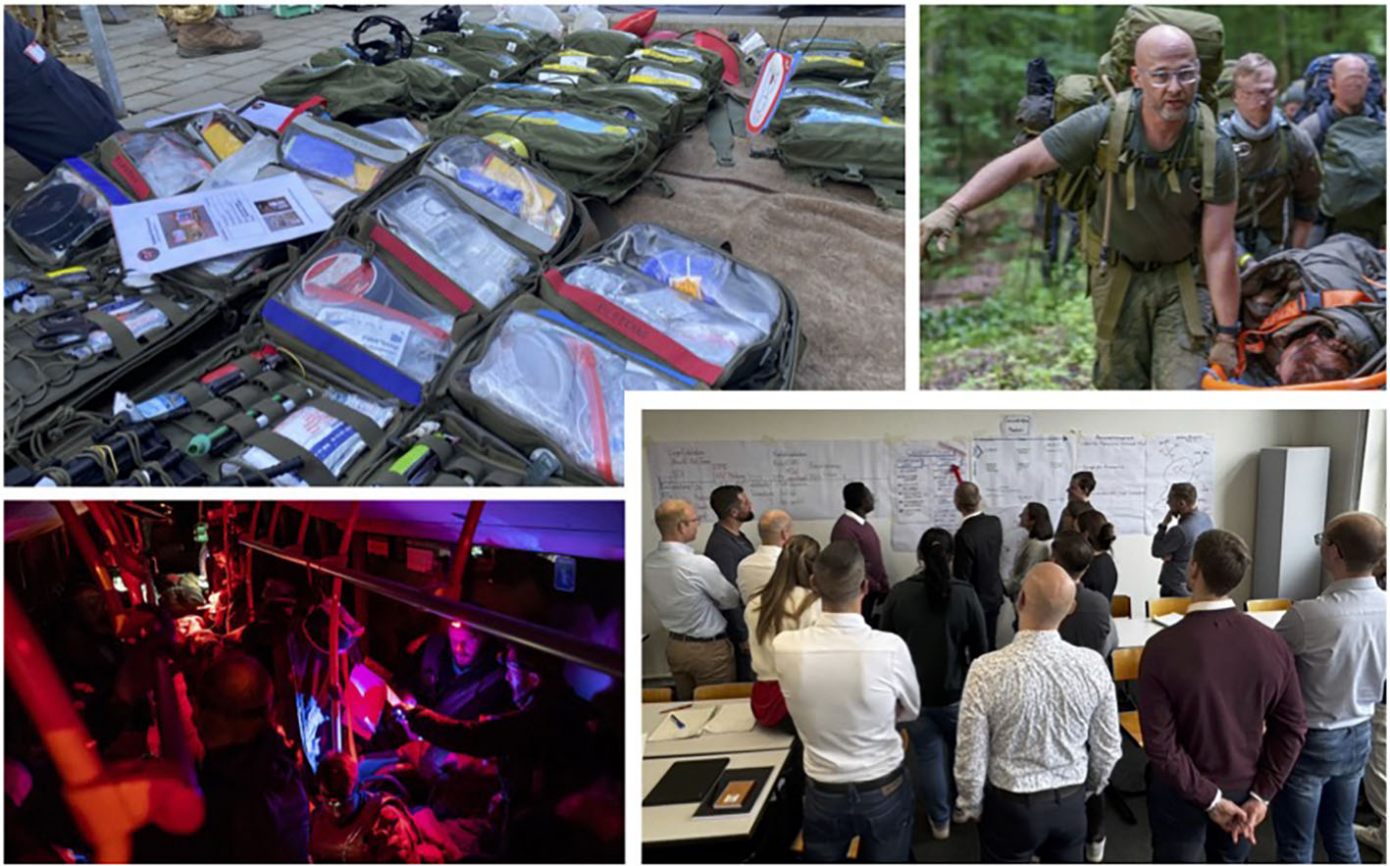
Tactical, medical, leading and SERE-elements of the curriculum. ^©^ BZGK.

Enabling the standardization of the various qualifications and to base them on the international qualifications framework, the degree program in Tactical Emergency Medicine for Disaster Management and Counterterrorism (Master Medic/Master Physician) was designed at the Department of Disaster Prevention and Crisis Management (BZGK) at University of Applied Sciences Fresenius in Idstein, Germany, with the title of Master Medic (non-medical personnel) or Master Physician (medical personnel) (22). It brings together civilian, police, and military expertise and train both medical-tactical and medical-operational leaders who can adequately manage special life-threatening operational situations with the appliance of the expertise of other stakeholders. This includes content such as leadership in special operational situations, including in crisis management teams, advanced medical care measures in the acute and prolonged care of severely injured patients, and technical rescue, including emergency decontamination and self-protection in medical chemical, biological, radiological, nuclear events like “CBRN” as well as scientific work in the context of disaster prevention and continuing study in pedagogic knowledge to be able to empower others. Some of these elements are shown in **Figure 1**. This content results from the integration of existing knowledge from the literature and expert knowledge from various sectors of emergency response, as well as the already established offerings of the Department of Disaster Prevention and Crisis Management (23, 24). It has been shown that case-based training, for example, is a key factor in improving situation management (25), especially when tailored to the needs of the individuals (26). At the same time there is also potential because of interaction and collaboration of the various actors (27).

Additionally, a survey was conducted among the target group. Convenience sampling was used to reach out at the target group. Information was spread via social media and gatekeeper from the personal and expanded network of the BZGK. Inclusion criteria were the profession in a medic sector like medicine, paramedic or military and police entrusted with medical tasks. A total of 80 people participated in the survey, of which 70 completed the entire questionnaire. Answering was anonymous. The sample included 4 women and 66 men. 68 of them gave information to carry a weapon. More than 50% (n=57) were Medics like SE Medics (n=36), Paramedics (n=15) and Emergency Paramedics (n=6). Additionally, professionals like physicians (n=2), Combat First Responders A/B, 18D or nursery staff took part in the survey. Participants were asked via online questionnaire whether a uniform curriculum for the Master Medic program was needed and what content should be taught. As a brief survey the questionnaire included some demographic data like qualification, use of weapons, gender and age and 5 more questions referring to the research topic. The data was analysed using descriptive statistics like statistical frequencies or qualitative content analysis for free field responses. 81% of respondents supported a uniform curriculum. The most prominent topics selected by the majority of respondents were:

- Mass casualty incidents & Triage Systems (97%)
- Advanced Trauma Life Support (63%)
- Leadership under stress (59%)
- Tactical Casualty Care (79%) and Prolonged Field Care (57%)

Most of the participants (n=32) decided to have a nearly equivalent relationship (50:50 or 40:60 or 60:40) between leading and medical skills.

At the end of the survey, participants had the opportunity to provide further important information. These included references to scenario training, legitimacy of the Master Medic within the various units, and the ability to empower other medics in special operational situations.

A special feature for imparting knowledge and skills is the teaching method of problem-oriented handling, which is a further development of problem-oriented learning (28). In the Tactical Emergency Medicine for Disaster Management and Counterterrorism (Master Medic/Master Physician) program, the content of one single semester is taught in a case study, which develops further over the entire semester and is worked through independently by the students under the supervision of the lecturers. This promotes collegial cooperation and independent problem-solving skills. In addition, students practice the theoretical and practical knowledge imparted, as well as the associated skills, in scenario-based simulation training, which is conducted by personnel with operational experience. This has proven to be a beneficial method in training (29). The students are mentally placed in an operational situation, which they work on in a team and with external participants. Feedback, briefing, and debriefing play an important role in professional reflection (30). By combining various teaching content and methods, the physical, cognitive, and psychological levels formulated in the Strategic Resilience model (6) are addressed, to positively influence human performance and thus the operational outcome. For example, there is the course 18F (24), which is included in the curriculum in the second semester. Students have some preparing lectures in advanced medical treatment, tactics and medical defends, which they must improve during the 18F. It is a longtime-scenario-simulation-approach. Students are mentally placed in a fictive foreign location where they have to build up the medical care under severe circumstances because of the locals and the overall security setting. Role players and realistic emergency personnel lead to scenarios as realistic as possible. The students live in the scenario for 3 days and must perform 5 scenarios, which took place after a short period of rest and partially at night without announcement. Furthermore, the level of difficulty gains weight. The students must decide and perform TCC or ALS in low regeneration-levels under time pressure by facing different challenges like mass casualty incidents, inappropriate material approach, CBRN or blackout. To support and evaluate the students professional staff is aside to coach nearly 1:1. Every scenario ends with a concrete debriefing to open the possibility of growing and empowering the human performance in situations that demand strategic resilience.

**Figure 2:**
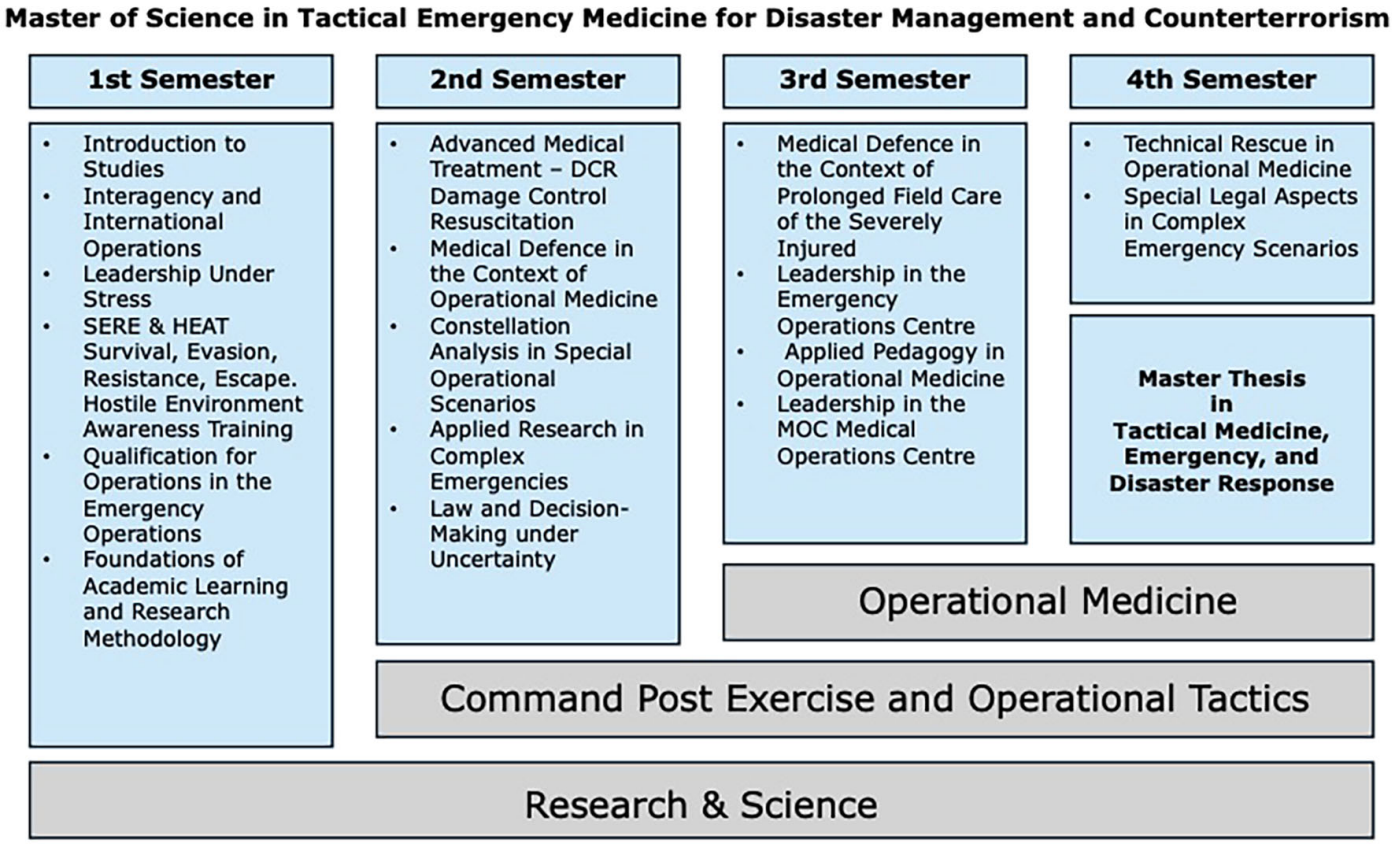
Content of the Master Medic/Master Physician program: Tactical Emergency Medicine for Disaster Management and Counterterrorism.

The program is designed to be part-time, allowing students to pursue regular employment and, as far as possible, apply the acquired knowledge to their everyday work. Due to the high proportion of practical instruction, the majority of teaching is in-person. The content of each semester is shown in **Figure 2**. It is supplemented by virtual lectures where possible and appropriate. The program lasts a total of four semesters. It is fully accredited at University of Applied Sciences Fresenius in Idstein, Germany, whose programs fit the benchmarks of the German Science and Humanities Council and the German Accreditation Council. The program began firstly in March 2025 and promises to meet the demands of a multidimensional education through the diverse professions of its students, including civilian healthcare, police and military emergency services, and non-police emergency services.

## 5 Conclusion

The diverse expertise of those involved in particularly life-threatening operational situations all makes an important contribution to managing such situations. The Master Medic/Master Physician program in Tactical Emergency Medicine for Disaster Management and Counterterrorism at the Department of Disaster Prevention and Crisis Management combines leadership, operational medicine, training and continuing education, as well as science and development. This provides the Master Medic/Master Physician with a comprehensive overview and the ability to act effectively. The program focuses on practical teaching and training using problem-oriented handling methods and simulations, which are facilitated by experienced police and non-police emergency response personnel. This ultimately means greater benefits for the protection of people, society, and cultural assets and meets the requirement of establishing and standardizing (NATO countries) a professional profile that is not yet defined but has become established as a concept. Transfer to other countries than Germany is not possible yet because the lectures are held in German. In future cooperation with other institutions or transfer in English is conceivable but limited due to different political circumstances or regularities.

## Data Availability

The raw data supporting the conclusions of this article will be made available by the authors, without undue reservation.
